# Supplementary material for: Interfering TAL effectors of Xanthomonas oryzae neutralize R-gene-mediated plant disease resistance
Source: Nat Commun. 2016 Nov 4;7:13435. doi: 10.1038/ncomms13435 (PMC5097170; doi:10.1038/ncomms13435)
Supplement: Supplementary Information — Supplementary Figure 1-18, Supplementary Tables 1-4 and Supplementary References. [file ncomms13435-s1.pdf]

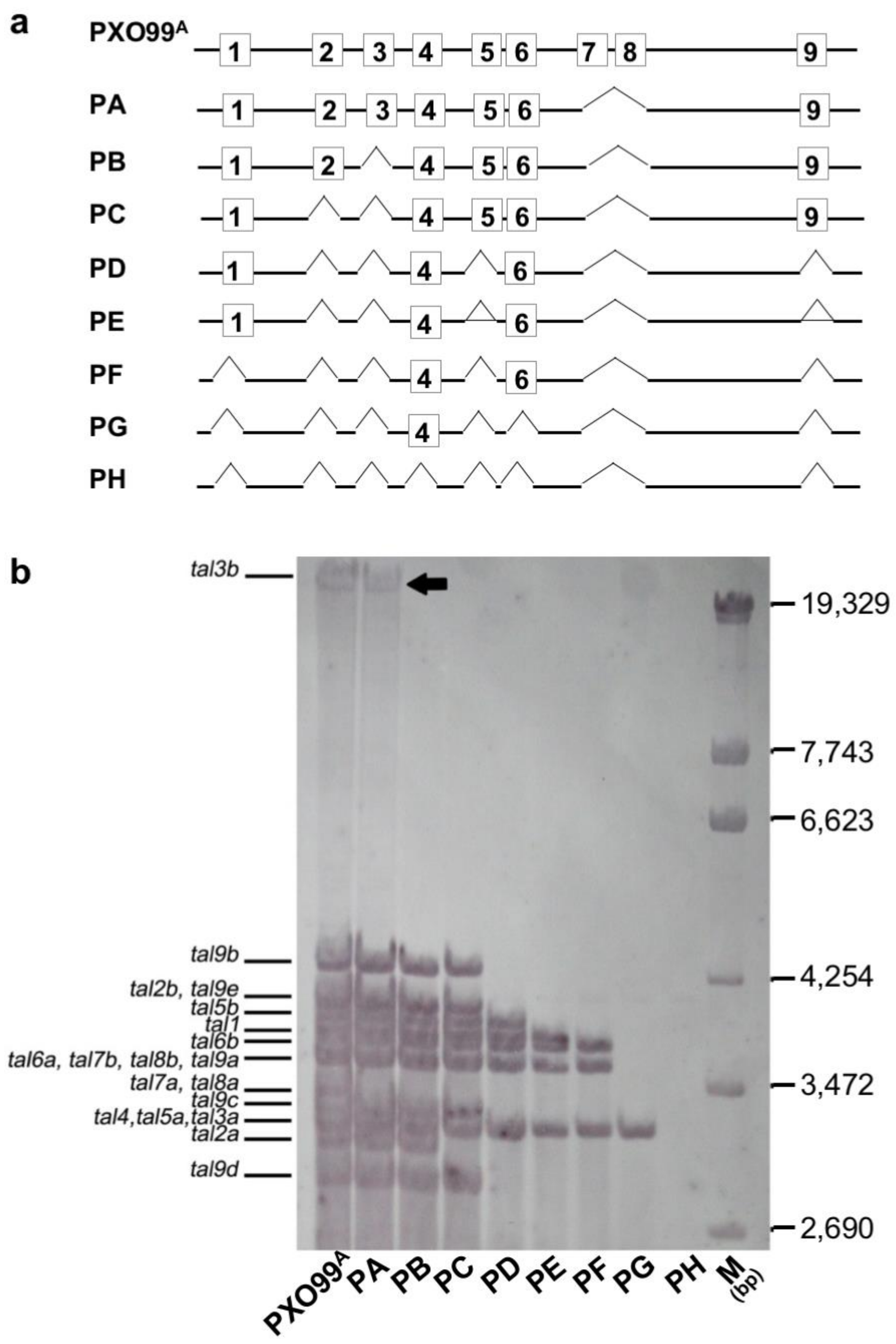

Supplementary Figure 1: TALE gene cluster deletions in PXO99^A. (**a**) Schematic diagrams show nine TALE gene clusters in PXO99^A and cluster deletions in mutants. Mutants (PA to PH) are sequential deletions of individual clusters as denoted by carets (^). (**b**) Southern blotting analysis of the TALE gene cluster mutants. After 8 rounds of sequential deletions, eight TALE mutants (PA to PH) including the TALE-free mutant PH were obtained. DNA ladder was denoted at the right side and TALE genes corresponding to the bands were marked at the left side.

A

```
        1   2   3    4   5   6   7   8   9  10  11  12  13  14  15  16  17  18
Tal3a  NS  HD  NG  NG  NG  HD  HD  NG  HD  NN  NG  HD  NN  DH  NG  HD  NI  N*
Tal3b  NS  HD  NG  NG  NG  NG  NG  HD  HD  HD  NN  HD  NG  HD  HD  HD  HD  N*
```

B

```
                    3813 bp                          4122 bp
              K  R  S  R| S  D  R  A  V                 |
pthXo1       AAACGGTCCCGATCGGATCGTGCTGTC.....TGA
             ********* ****************
Tal3a        AAACGGTCCTGATCGGATCGTGCTGTC
              K  R  S  *
```

C

```
                       3360 bp
          G  R  P  A  P|...A  F  N  E  E  E  L  A  W  L ....
pthXo1   GGACGTCCTGCCCCG...CATTCAACGAAGAGGAGCTCGCATGGTTGA...
         ***************   ******* *******    ******* ****
Tal3b    GGACGTCCTGCCCCG^CATTCAAGGAAGAGGAAATCGCATGATTGA
          G  R  P  A  P     H  S  R  K  R  K  S  H  D  *
```

D

```
>PthXo1 C-terminus
SIVAQLSRPDPALAALTNDHLVALACLGGRPAMDAVKKGLPHAPELIRRVNRRIGE
RTSHRVADYAQVVRVLEFFQCHSHPAYAFDEAMTQFGMSRNGLVQLFRRVGVTELE
ARGGTLPPASQRWDRILQASGMKRAKPSPTSAQTPDQASLHAFADSLERDLDAPSP
MHEGDQTGASSRKRSRSDRAVTGPSAQHSFEVRVPEQRDALHLPLSWRVKRPRTRI
GGGLPDPGTPIAADLAASSTVMWEQDAAPFAGAADDFPAFNEEELAWLMELLPQSG
SVGGTI
>Tal3a C-terminus
SIVAQLSRPDPALAALTNDHLVALACLGGRPALDAVKKGLPHAPELIRRVNSRIGE
RTSHRVADLAHVVRVLGFFQSHSHPAQAFDDAMTQFGMSRHGLVQLFRRVGVTEFE
ARCGTIPPASQRWDRILQASGTKRAKPSPTSAQTPDQASLHAFPDSLERDLDAPSP
MHEGDQTRASRRKRS
>Tal3b C-terminus
SIVAQLSRRDPALAALTNDQLVALACLGGRPAPHSRKRKSHD
```

Supplementary Figure 2: Sequence information of Tal3a and Tal3b. (**a**) The central repeats of predicted Tal3a and Tal3b in order of RVD (repeat variable di-amino acids). Underlined RVDs represent a repeat with a unique length of 28 amino acids compared to normal 34 amino acids. An asterisk (*) denotes the last half repeat. (**b**) Partial sequences of *Tal3a* and the predicted truncated effector relative to *pthXo1*. (**c**) Partial sequences of *Tal3b* and the predicted truncated effector relative to *pthXo1*. (**d**) Predicted amino acid sequences of the C-termini of Tal3a and Tal3b compared to PthXo1. Underlined sequences are identical for the three TALEs; letters in red are predicted nuclear localization signals; bold letters are C-terminal transcription activation domain in PthXo1 but truncated in Tal3a and Tal3b.

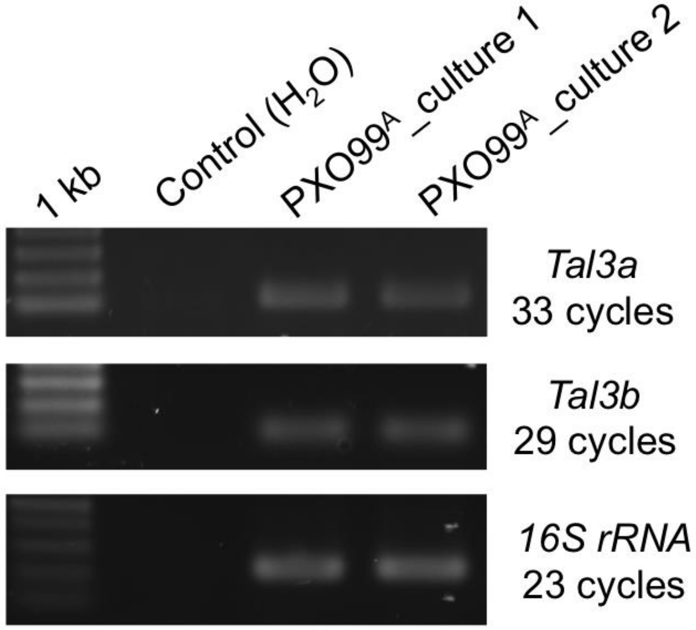

Supplementary Figure 3: iTALE genes *Tal3a* and *Tal3b* were expressed in bacteria. RNA from two PXO99^A cultures (as indicated above each lane) was used for cDNA synthesis and PCR with gene specific primers. 16S rRNA gene expression was used as an internal control.

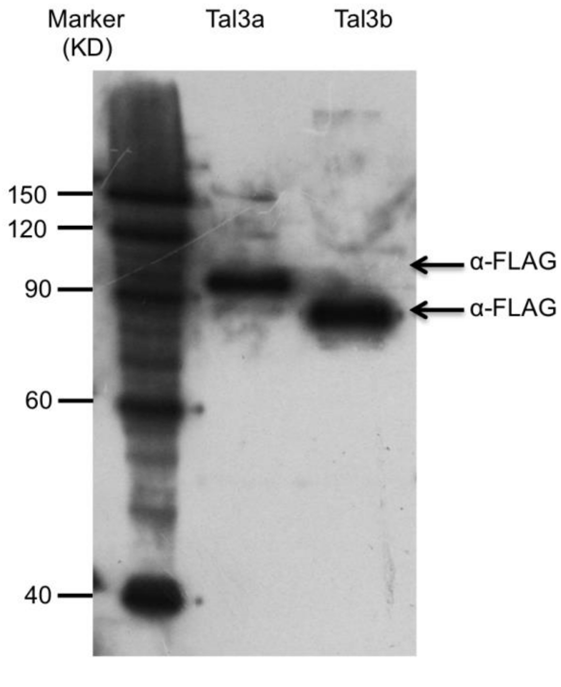

Supplementary Figure 4: Western blotting analysis of Tal3a and Tal3b. Bacterial extract was separated through gel electrophoresis and blotted/probed with the monoclonal antibody against the epitope FLAG.

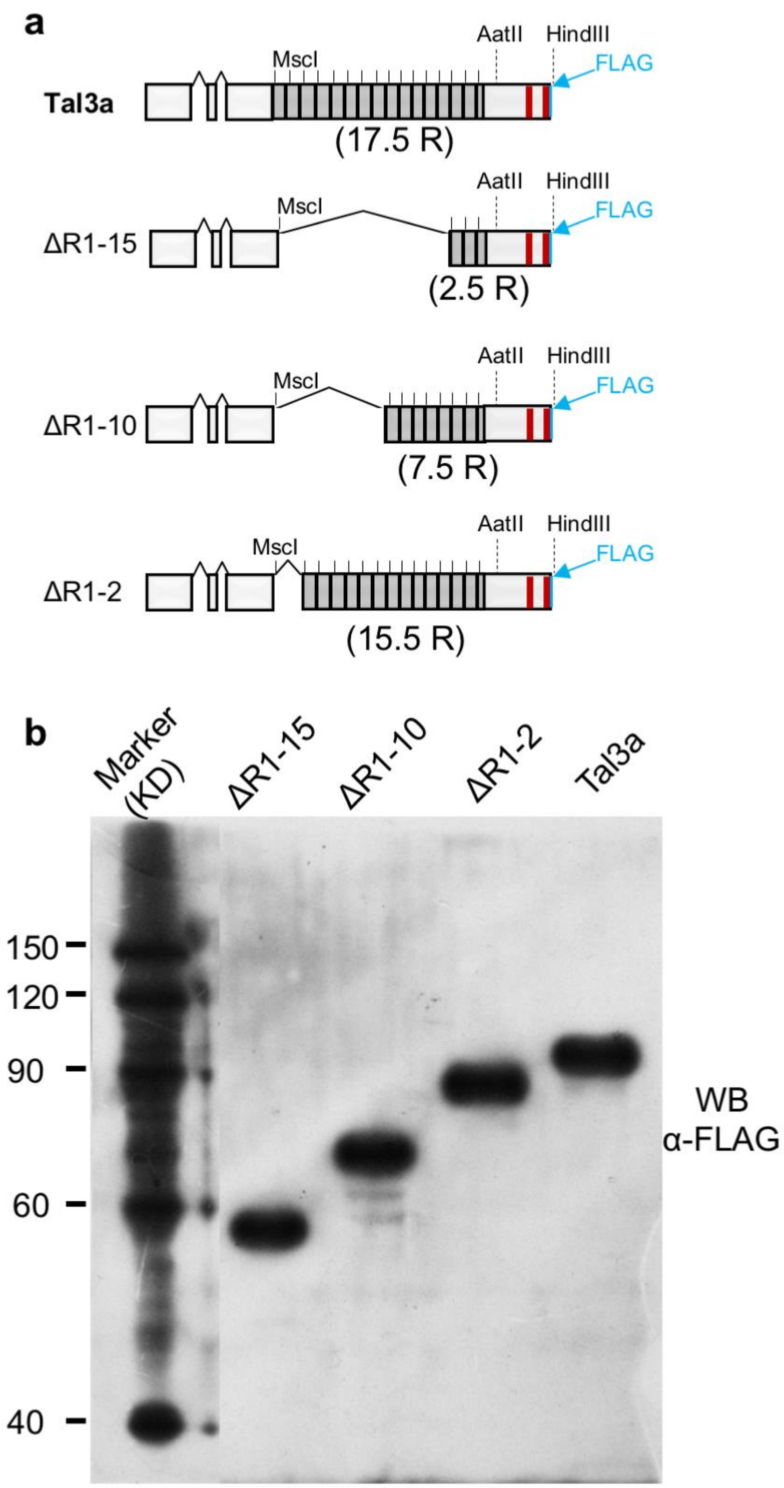

Supplementary Figure 5: Western blotting analysis of Tal3a and its repeat deletion mutants. (**a**) Schematic diagrams show structures of Tal3a and its derivatives. Genes with internal repeat deletions were obtained by partially deleting MscI fragments at the 5' ends. (**b**) Western blotting analysis. Bacterial extract was separated through gel electrophoresis and blotted/probed with monoclonal antibody against epitope FLAG.

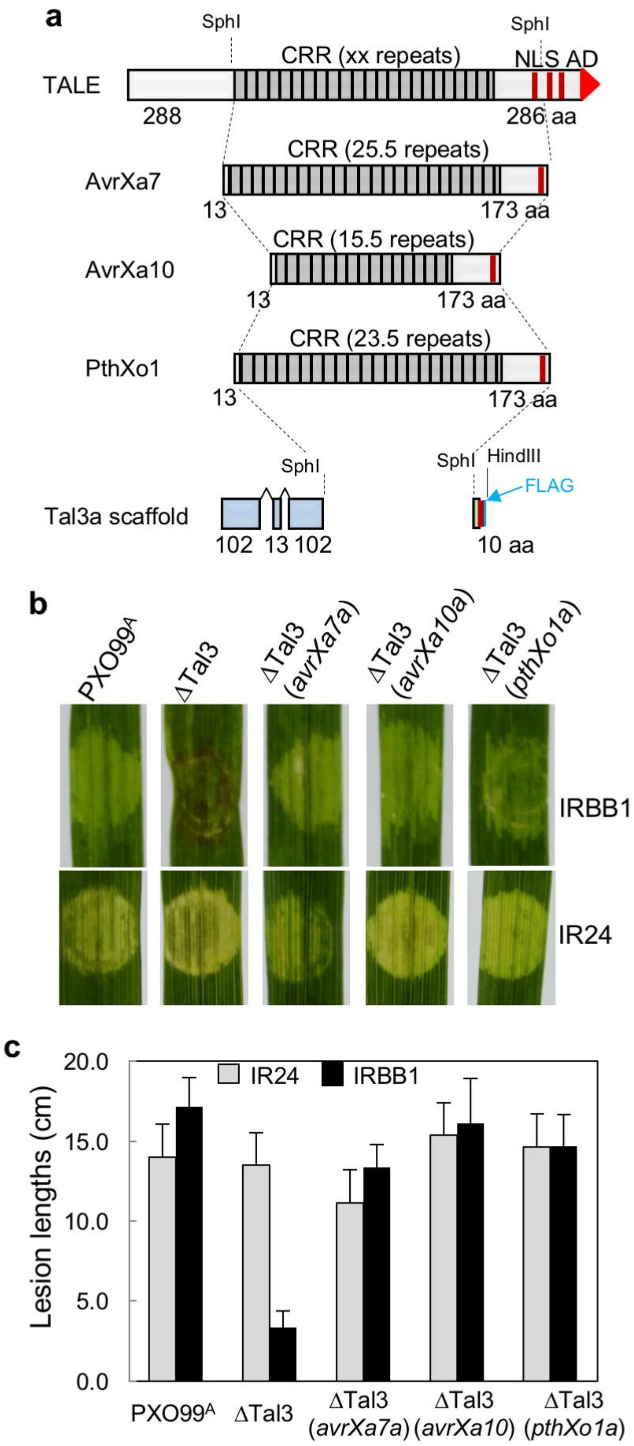

Supplementary Figure 6: TAL effectors containing repeats of various TAL effector genes in the Tal3a scaffold function as resistance suppressors. (**a**) Schematic diagrams showing how *avrXa7a*, *avrXa10* and *pthXo1* were constructed. SphI fragments in the full-length TALE genes were swapped into the corresponding region of the repeat-less *Tal3a*. (**b**) Disease reactions of IR24 and IRBB1 in response to various Xoo strains. Photos were taken 2 days post inoculation with clearing color indicating disease reaction and brown color denoting HR. (**c**) Lesion lengths caused by different strains in IR24 and IRBB1. Error bars (standard deviation, n=10, 3 replicates) with the same letter do not differ from each other at $p<0.05$ (Tukey test).

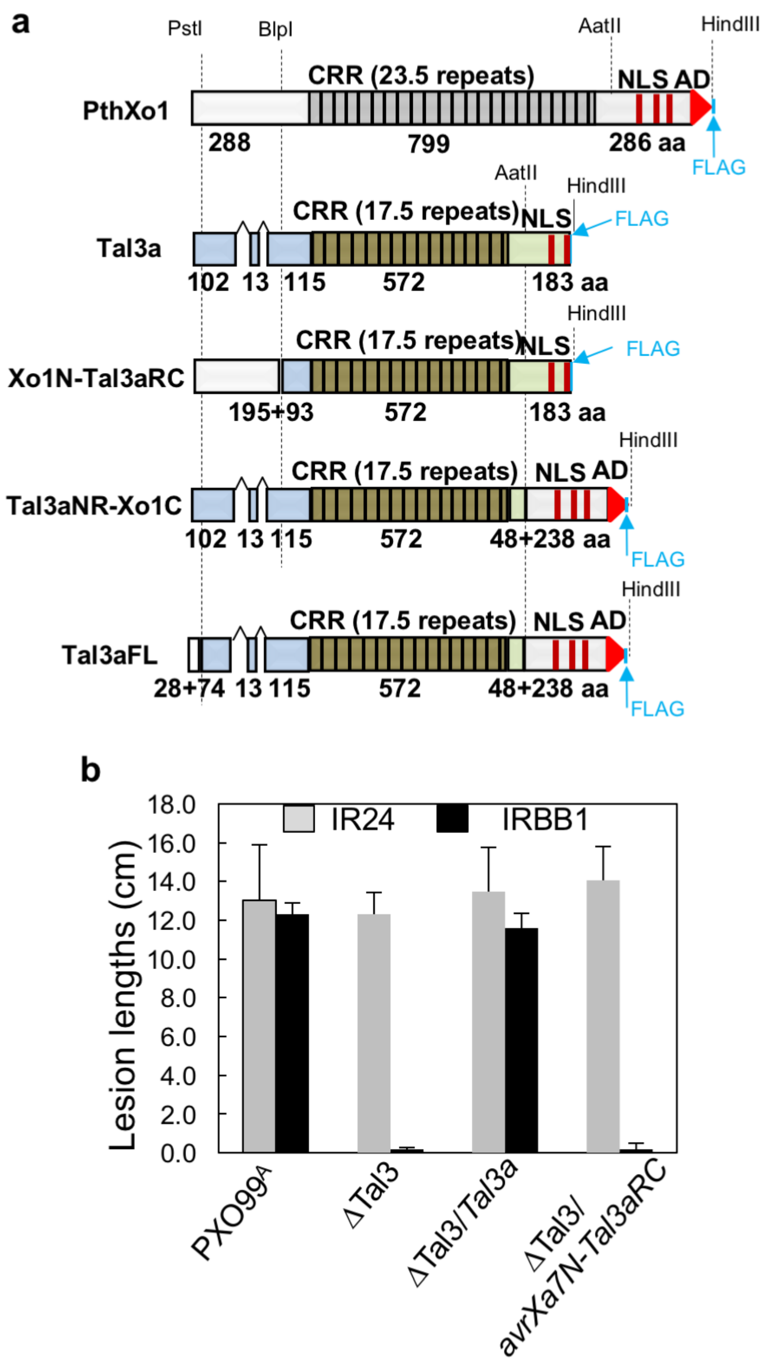

Supplementary Figure 7: N-terminal domain of Tal3a is required for its ability to suppress resistance to ΔTal3 in IRBB1. (**a**) Schematic diagrams show how hybrids of TALEs were made. Fragment defined by PstI and BlpI cleavage sites from *pthXo1* was swapped into *Tal3a*, resulting in Xo1N-Tal3aRC; AatII and HindIII fragment from *pthXo1* was swapped into *Tal3a* to produce Tal3aNR-Xo1C; and finally PstI-AatII fragment from *Tal3a* was swapped into *pthXo1*, resulting in Tal3aFL. (**b**) The Xoo strains were indicated below each column. *avrXa7N-Tal3aRC* is a chimeric gene of AvrXa7 N-terminus coding region and *Tal3a* central and C-terminal domain coding regions. Lesion lengths were measured at 14 DPI. Error bars (standard deviation, n=10, 3 replicates) with the same letter do not differ from each other at $p<0.05$ (Tukey test).

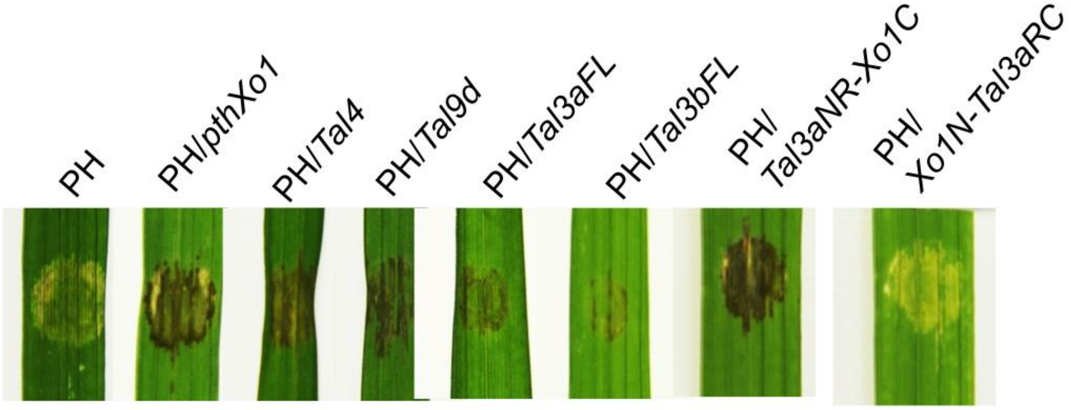

Supplementary Figure 8: HR to various full-length TALEs in *Xa1*-transgenic plants. Leaves of *Xa1* transgenic Kitaake were inoculated with PH strains containing individual TALE genes as indicated. PH is a PXO99^{A} TALE-free mutant. *pthXo1*, *Tal4* and *Tal9d* are three plasmid-borne TALE genes from PXO99^{A}. *Tal3aFL* and *Tal3bFL* are *avrXa7* variants swapped with *Tal3a* and *Tal3b* repetitive domains, resulting in the restructured full-length versions of *Tal3a* and *Tal3b*, respectively. *Tal3aNR-Xo1C* consists of Tal3a N-terminal and repetitive domains and PthXo1 C-terminus. Xo1N-Tal3aRC is a Tal3a variant containing PthXo1 N-terminus and Tal3a central repetitive and C-terminal domains. Photos were taken at 3 DPI.

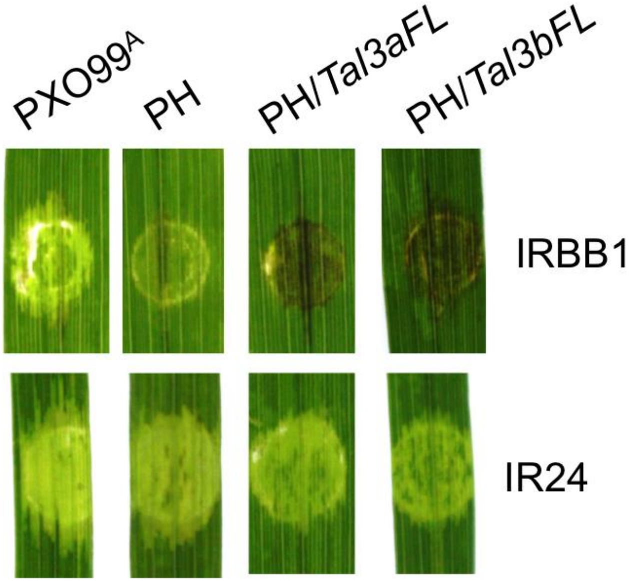

Supplementary Figure 9: Full-length C-terminus of TALE enabled Tal3a and Tal3b to trigger HR specifically in IRBB1. The Xoo strains were indicated above the leaves. PH is the TALE gene free mutant of PXO99^{A}. Photos were taken at 3 DPI.

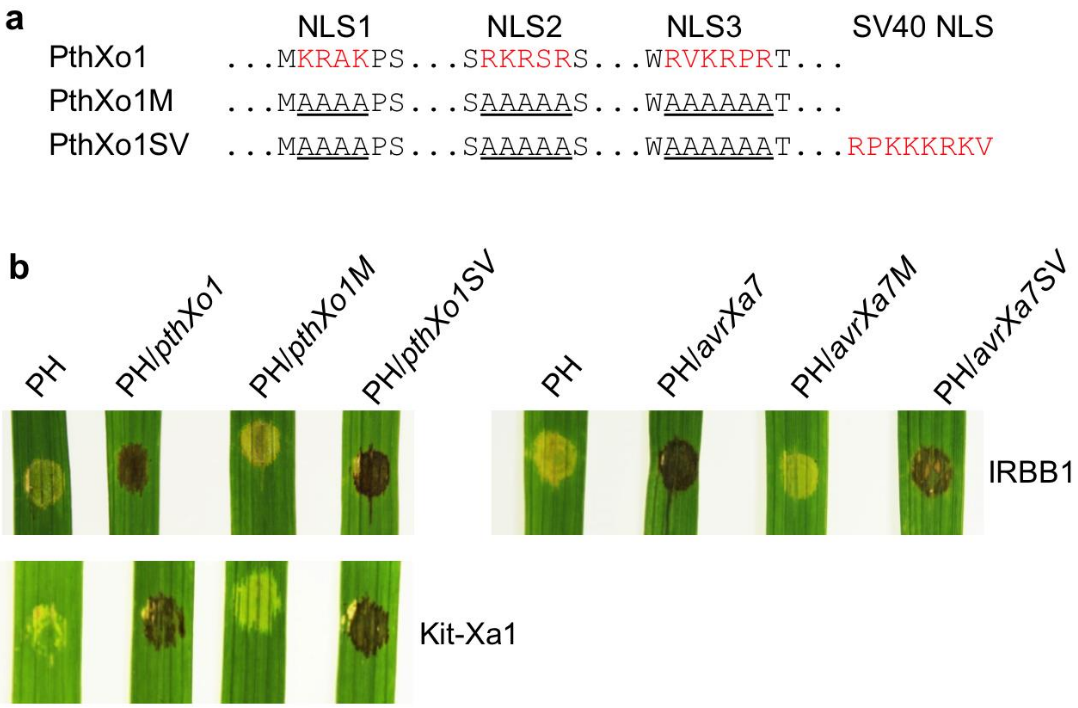

Supplementary Figure 10: Nuclear localization motifs in PthXo1 and AvrXa7 are required for activation of *Xa1* resistance. (**a**) Amino acids of the NLS and replacements in PthXo1 and its derivatives are in red and underlined. Sequences for AvrXa7 and its derivatives were as described in a prior study (ref. 1). (**b**) Disease reactions in *Xa1* containing plants to various Xoo strains as indicated above the leaves. Photos were taken 3 DPI.

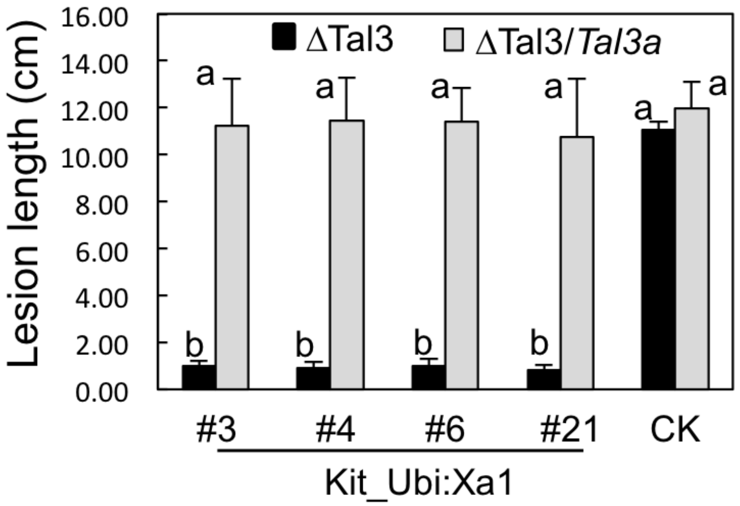

Supplementary Figure 11: Overexpressed *Xa1* confers resistance to ΔTal3 and iTALE gene *Tal3a* suppresses the resistance. Four lines of transgenic Kitaake expressing *Xa1* under the rice ubiquitin promoter were resistant to ΔTal3 strain but susceptible to the complementing strain ΔTal3 with *Tal3a*. The non-transgenic plants (CK) were susceptible to both ΔTal3 and ΔTal3/*Tal3a*. Lesion lengths were measured 12 DPI. Error bars (standard deviation, n=8, 2 replicates) with the same letter do not differ from each other at $p<0.05$ (Tukey test).

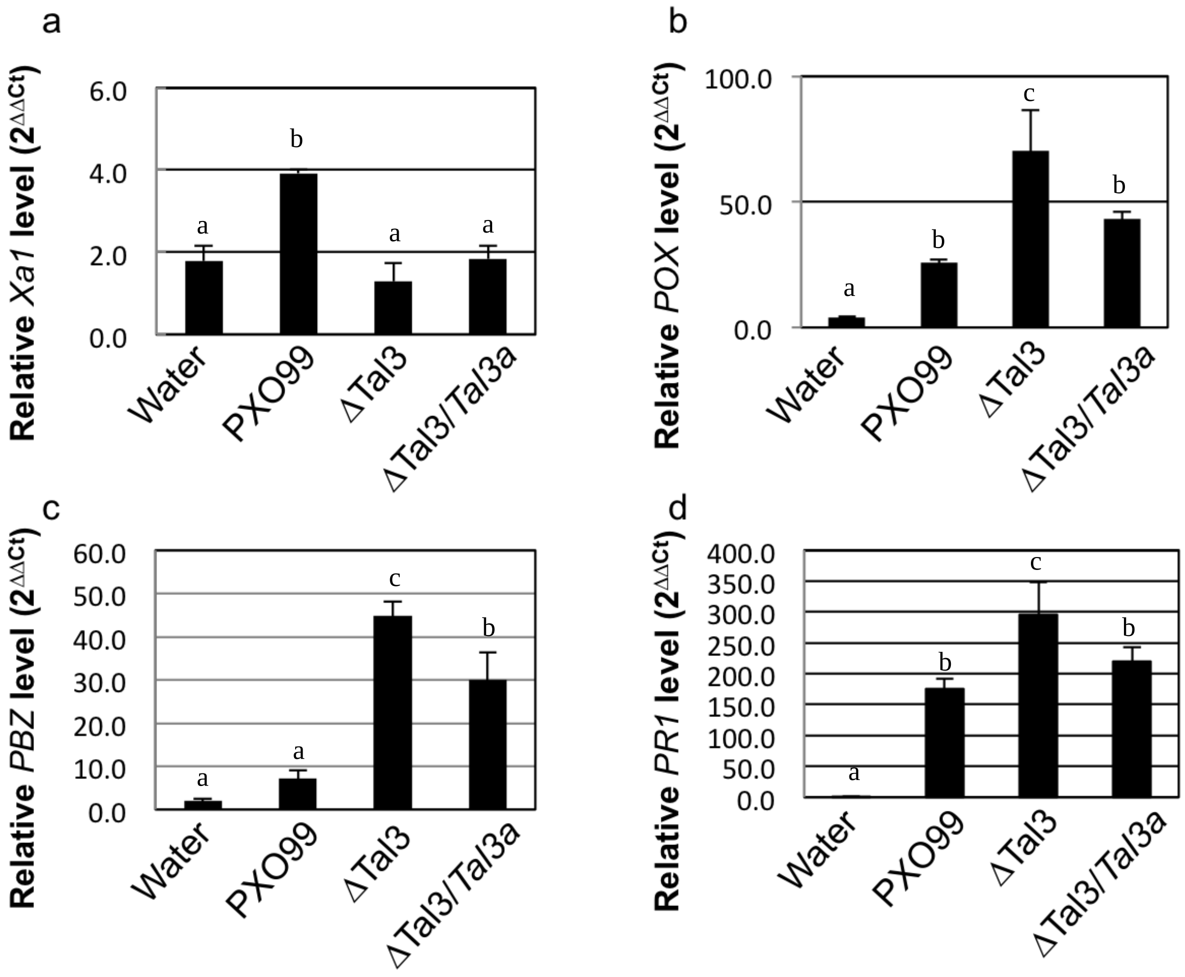

Supplementary Figure 12: Rice defense genes in IRBB1 are induced by TALE and the gene induction is suppressed by Tal3a. Three-weeks-old plants of IRBB1were inoculated with water (wounding), PXO99^{A}, ΔTal3 and ΔTal3/*Tal3a*, and total RNA was isolated at 24 hr after inoculation. cDNA prepared from the samples was subjected to quantitative RT-PCR using primers specific to *Xa1* (**a**), peroxidase *PXO22.3* (Os07g48020) (**b**), *PBZ* (probenazole-inducible gene, Os12g36880) (**c**) and *PR1* (pathogenesis-related gene, Os07g03730) (**d**). Gene-specific primers for the rice actin gene (Os03g50885) were used for control. The expression level relative to that of non-inoculated leaves is presented in average threshold cycle (Ct) using the $2^{-\Delta\Delta Ct}$ method (ref. 2). Error bars (standard deviation from 3 replicates) with the same letter do not differ from each other at $p<0.05$ (Tukey test).

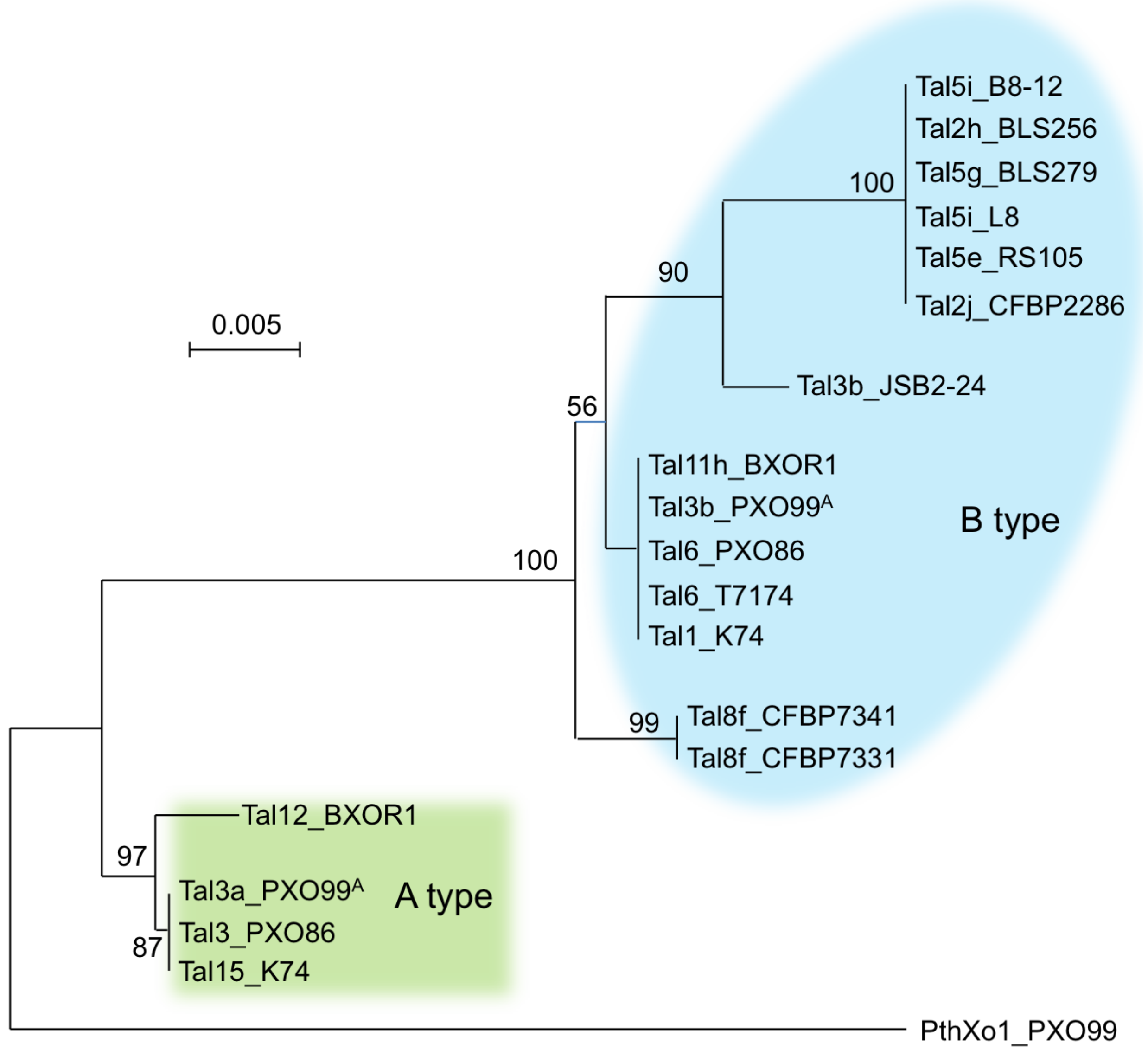

Supplementary Figure 13: Unrooted phylogenic tree of 18 iTALE genes from Xoo (n=4) and Xoc (n=14). Bootstrap values shown at nodes were obtained from 1,000 trials, and branch lengths correspond to the divergence of DNA sequences, as indicated by the relative scale. Neighbor-Joining method (Software Mega 6, http://www.megasoftware.net) was used for the tree.

a

SIVAQLSRPDPALAALTNDHLVALACLGGRPALDAVKKGLPHAPELIRRVNSRIGER
TSHRVADLAHVVRVLGFFQSHSHPAQAFDDAMTQFGMSRHGLVQLFRRVGVTEFEAR
CGTIPPASQRWDRILQASGTKRAKPSPTSAQTPDQASLHAFPDSLERDLDAPSPMHE
GDQTRASRRKRS

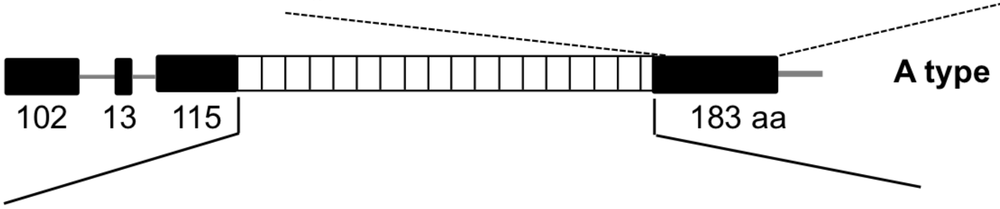

	1	2	3	4	5	6	7	8	9	10	11	12	13	14	15	16	17	18	19
X. o.* pv. *oryzae																			
Tal3a_PXO99^{A}	NS	HD	NG	NG	NG	HD	HD	NG	HD	NN	NG	HD	NN	DH	NG	HD	NI	N*	
Tal3_PXO86	NS	HD	NG	NG	NG	NG	NG	HD	HD	HD	NN	HD	NG	HD	NI	HD	NN	N*	
Tal15_K74	NS	HD	NG	NG	NG	NG	HD	HD	HD	HD	NN	HD	NG	HD	NI	HD	NN	N*	
X. o.* pv. *oryzicola																			
Tal12_BXOR1	NI	HG	HG	HG	HG	HG	NG	HD	HD	HD	HD	HD	NG	NG	NI	NN	HD	HD	H*

b

SIVAQLSRRDPALAALTKDQLVALACFGGRPAPHSRKRKSHD

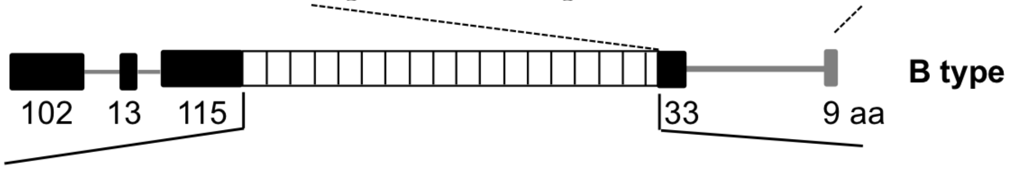

	1	2	3	4	5	6	7	8	9	10	11	12	13	14	15	16	17	18	19	20	21	22	23	24
X. o.* pv. *oryzae																								
Tal3b_PXO99^{A}	NS	HD	NG	NG	NG	NG	NG	HD	HD	HD	NN	HD	NG	HD	HD	HD	HD	N*						
Tal6_PXO86	NS	NG	NG	NG	NG	NG	NG	HD	HD	HD	NN	HD	NG	HD	HD	HD	HD	H*						
Tal6_T7174	NS	HD	NG	NG	NG	NG	HD	HD	HD	HD	NN	HD	NG	HD	HD	HD	NN	H*						
Tal1_K74	NS	HD	NG	NG	NG	NG	HD	HD	HD	HD	NN	HD	HD	HD	HD	NN	H*							
X. o.* pv. *oryzicola																								
Tal5i_B8-12	NI	ND	HG	HG	HG	HG	NG	HG	HG	HD	HD	HD	HD	HD	NN	NN	HD	NG	HH	H*	H*	NN	HD	H*
Tal5e_RS105	NI	ND	HG	HG	HG	HG	NG	HG	HG	HD	HD	HD	HD	NN	NN	HD	HG	HH	H*	H*	NN	HD	H*	
Tal5i_L8	NI	HD	HG	HG	HG	NG	HG	HG	HD	HD	HD	NN	NN	HD	HG	HH	H*	H*	NN	HD	H*			
Tal2j_CFBP2286	NS	ND	HG	HG	HG	NG	HG	HD	HD	HD	HD	NN	NN	HD	HG	HH	H*	H*	NN	HD	H*			
Tal3b_JSB2-24	NI	HG	HG	HG	HG	HG	NG	HD	HD	HD	NN	NN	HD	HG	HH	HG	HG	NN	HD	H*				
Tal5g_BLS279	NI	ND	HG	HG	HG	NI	HG	HG	HD	HD	HD	NN	NN	HD	HG	HH	N*	H*	NN	HD	H*			
Tal2h_BLS256	NS	ND	HG	HG	HG	NG	HG	HG	HD	HD	HD	NN	NN	HD	HG	HH	H*	H*	NN	HD	H*			
Tal11h_BXOR1	NI	HG	HG	HG	HG	HG	NG	HD	HD	HD	HD	HD	NG	NG	NI	NN	HD	HD	H*					
Tal8f_CFBP7331	NI	HD	HD	N*	HD	H*																		
Tal8f_CFBP7341	NI	HD	HD	N*	HD	H*																		

Supplementary Figure 14: Alignment of repeats of 18 iTALEs as represented by the 12th and 13th amino acids (RVD) of each repeat. The single amino acid code is used for each amino residue. An asterisk (*) denotes the missing 13th amino acid. Underlined RVD represents a repeat with a deletion of 6 amino acids (23th to 28th). (**a**) and (**b**) represent type A and type B effectors, respectively.

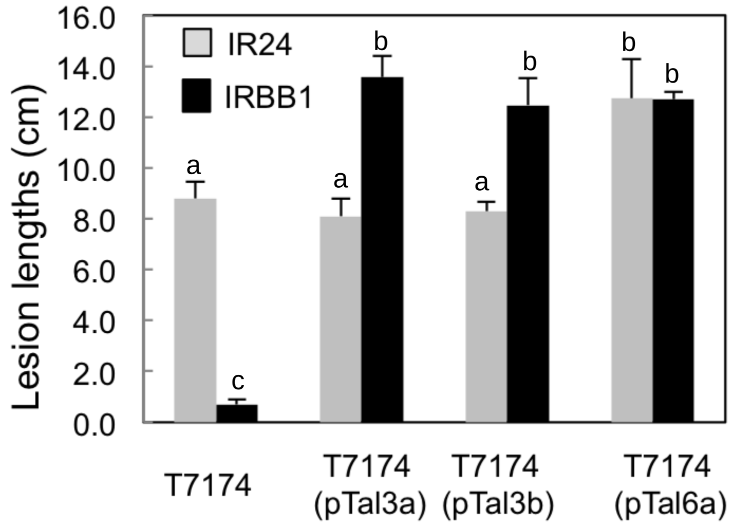

Supplementary Figure 15: iTALEs enable IRBB1-imcompatible strain T7174 compatible to IRBB1. Xoo strains with the plasmid-borne *Tal3a* and *Tal3b* from PXO99^{A} and *Tal6a* from T7174 in the scaffold of *Tal3a* were inoculated in IR24 (T7174 compatible) and IRBB1. Lesion lengths were measured 12 days post inoculation. Error bars (standard deviation, n=10, 3 replicates) with the same letter do not differ from each other at p<0.05 (Tukey test).

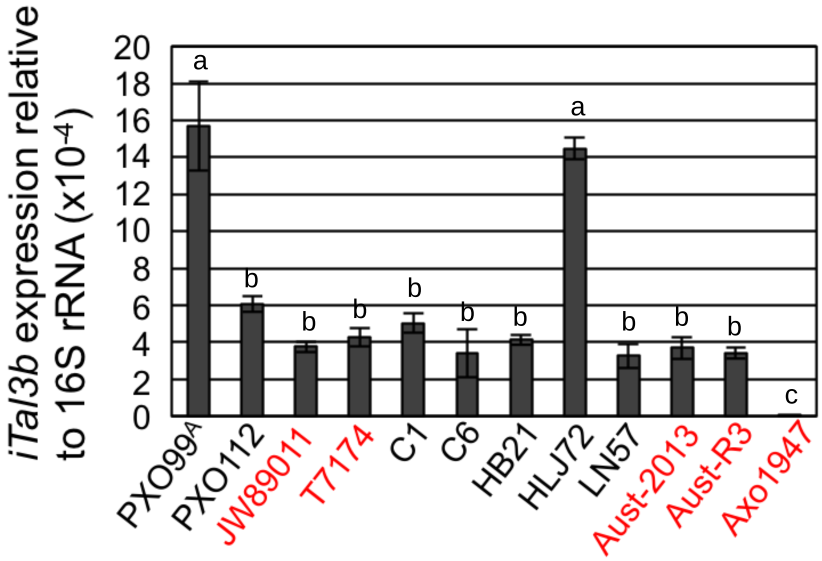

Supplementary Figure 16: expression of type B iTALE genes in different Xoo strains grown in medium. Quantitative RT-PCR was performed on bacterial total RNA with expression levels of type B genes relative to the 16S rRNA gene. Strains are indicated below individual bars and the IRBB1-incompatible strains are labeled in red. All strains except PXO99^{A} and AXO1947 contain only type B iTALE gene. Error bars represent standard deviation of three replicates. Bars with the same letter different from each other at p<0.05 (Tukey test).

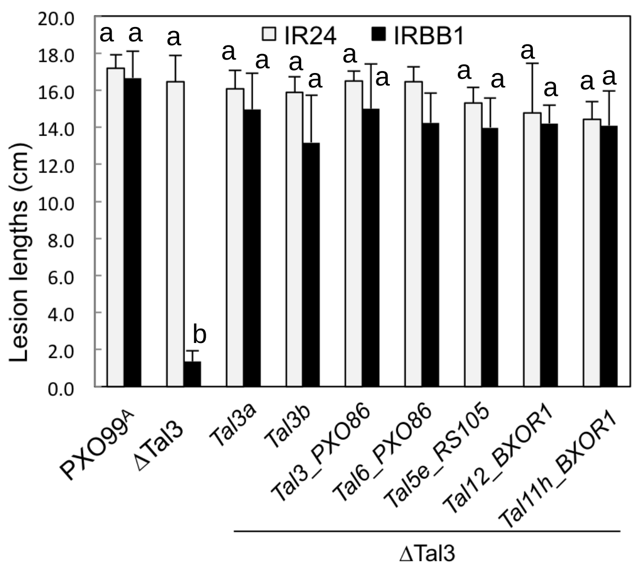

Supplementary Figure 17: iTALEs from Xoo and Xoc suppress ΔTal3 triggered resistance in IRBB1. Five-week old IR24 and IRBB1 plants were inoculated with strains as indicated below each column using leaf-tip clipping method. Lesion lengths were measured at 13 DPI. Error bars (standard deviation, n=6, 2 replicates) with the same letter do not differ from each other at $p<0.05$ (Tukey test).

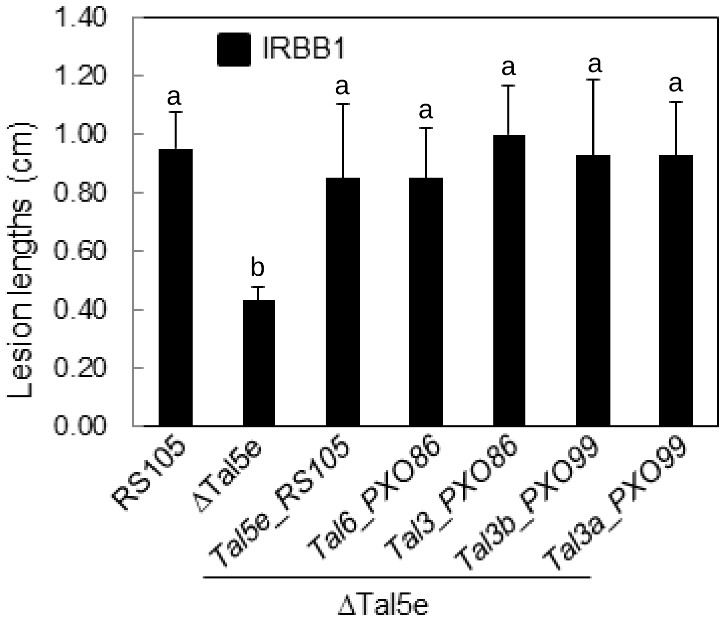

Supplementary Figure 18: Suppressive activity of the Xoo iTALE genes in Xoc. iTALE genes from PXO86 and PXO99^{A} of Xoo restore the Xoc mutant (ΔTal5e) ability to cause disease in IRBB1 in term of lesion length measured at 7 DPI. Error bars (standard deviation, n=6, 2 replicates) with the same letter do not differ from each other at $p<0.05$ (Tukey test).

Supplementary Table 1. Lesion lengths (cm) of different rice varieties caused by PXO99^{A} and its TALE gene deletion mutants.

Rice Lines	PXO99 and its TALE gene cluster Mutants								
	PXO99	PA	PB	PC	PD	PE	PF	PG	PH
IRBB1	13.3 ± 4.2^{A}	12.1 ± 3.2^{A}	$3.3.\pm1.1^{B}$	0.7 ± 0.6^{B}	0.4 ± 0.2^{B}	0.7 ± 0.5^{B}	1.2 ± 0.8^{B}	0.7 ± 0.4^{B}	1.2 ± 0.6^{B}
IRBB3	23.3 ± 2.9^{A}	26.7 ± 5.4^{A}	25.4 ± 4.5^{A}	1.9 ± 1.2^{B}	1.4 ± 1.8^{B}	1.1 ± 1.2^{B}	1.1 ± 1.2^{B}	0.8 ± 0.5^{B}	1.2 ± 1.0^{B}
IRBB4	23.2 ± 4.4^{A}	23.1 ± 3.2^{A}	22.3 ± 9.0^{A}	2.8 ± 1.4^{B}	2.2 ± 1.1^{B}	2.5 ± 2.0^{B}	1.2 ± 0.9^{B}	1.0 ± 0.5^{B}	0.7 ± 0.4^{B}
IRBB5	15.3 ± 3.4^{A}	15.9 ± 0.8^{A}	16.5 ± 2.7^{A}	1.1 ± 0.6^{B}	0.3 ± 0.3^{B}	0.5 ± 0.3^{B}	0.3 ± 0.2^{B}	0.6 ± 0.4^{B}	0.8 ± 0.6^{B}
IRBB7	7.0 ± 2.8^{A}	7.04 ± 2.6^{A}	5.5 ± 1.8^{A}	0.4 ± 0.4^{B}	0.3 ± 0.2^{B}	0.3 ± 0.2^{B}	0.6 ± 0.4^{B}	0.4 ± 0.3^{B}	0.3 ± 0.1^{B}
IRBB8	16.6 ± 1.5^{A}	15.5 ± 0.8^{A}	13.2 ± 2.2^{B}	1.3 ± 0.4^{C}	1.6 ± 0.6^{C}	1.5 ± 0.7^{C}	1.3 ± 0.2^{C}	2.2 ± 0.7^{C}	2.4 ± 0.2^{C}
IRBB10	20.5 ± 5.3^{A}	18.4 ± 5.2^{A}	17.6 ± 1.9^{A}	1.3 ± 0.4^{B}	0.5 ± 0.4^{B}	0.97 ± 0.5^{B}	0.4 ± 0.2^{B}	0.2 ± 0.1^{B}	0.5 ± 0.2^{B}
IRBB11	22.0 ± 3.1^{A}	21.3 ± 3.4^{A}	20.6 ± 4.5^{A}	2.3 ± 0.9^{B}	1.5 ± 0.2^{B}	1.8 ± 0.3^{B}	1.9 ± 0.4^{B}	2.0 ± 0.2^{B}	1.6 ± 0.3^{B}
IRBB11(T)	8.2 ± 2.5^{A}	8.1 ± 2.6^{A}	5.9 ± 3.2^{AB}	4.4 ± 3.4^{BC}	2.0 ± 1.0^{C}	1.1 ± 0.4^{C}	1.0 ± 0.5^{C}	1.4 ± 0.3^{C}	1.8 ± 0.8^{C}
IRBB13	1.8 ± 1.0^{A}	2.0 ± 1.0^{A}	1.0 ± 0.5^{AB}	1.1 ± 0.5^{AB}	0.9 ± 0.4^{AB}	0.4 ± 0.2^{B}	0.3 ± 0.1^{B}	0.4 ± 0.4^{B}	0.6 ± 0.5^{B}
IRBB21	1.6 ± 1.3^{A}	0.4 ± 0.3^{B}	0.4 ± 0.2^{B}	0.7 ± 0.4^{AB}	1.0 ± 0.3^{AB}	1.1 ± 0.4^{AB}	0.8 ± 0.2^{AB}	0.8 ± 0.2^{AB}	0.4 ± 0.1^{B}
IRBB50	14.1 ± 6.0^{A}	10.6 ± 3.3^{A}	11.8 ± 2.2^{A}	2.1 ± 0.4^{B}	1.2 ± 0.1^{B}	0.7 ± 0.3^{B}	1.2 ± 0.2^{B}	1.0 ± 0.5^{B}	1.2 ± 0.7^{B}
IRBB51	2.3 ± 0.8^{AB}	2.3 ± 0.7^{AB}	2.0 ± 0.5^{ABC}	3.1 ± 0.6^{A}	1.3 ± 0.3^{BCD}	1.2 ± 0.5^{BCD}	0.6 ± 0.3^{D}	0.5 ± 0.4^{D}	0.9 ± 0.6^{CD}
IRBB52	1.1 ± 0.7^{A}	1.0 ± 0.4^{AB}	0.9 ± 0.5^{AB}	1.2 ± 0.7^{A}	0.3 ± 0.2^{BC}	0.3 ± 0.1^{BC}	0.3 ± 0.2^{BC}	0.3 ± 0.2^{BC}	0.2 ± 0.1^{C}
IRBB53	0.7 ± 0.3^{AB}	0.5 ± 0.1^{AB}	0.7 ± 0.3^{AB}	0.5 ± 0.2^{AB}	0.4 ± 0.2^{AB}	0.5 ± 0.2^{AB}	0.5 ± 0.4^{AB}	0.3 ± 0.1^{B}	0.9 ± 0.5^{A}
IRBB54	1.8 ± 0.5^{ABC}	2.0 ± 1.0^{ABC}	1.6 ± 0.3^{ABCD}	2.2 ± 0.2^{AB}	0.6 ± 0.4^{BCD}	2.0 ± 1.8^{ABC}	2.8 ± 1.2^{A}	0.3 ± 0.1^{D}	0.4 ± 0.2^{CD}
IRBB55	4.14 ± 1.0^{A}	4.8 ± 1.80^{A}	1.95 ± 0.7^{B}	1.0 ± 0.4^{BC}	0.7 ± 0.5^{BC}	0.8 ± 0.4^{BC}	0.4 ± 0.2^{C}	0.3 ± 0.1^{C}	0.4 ± 0.2^{C}
IRBB203	26.2 ± 7.4^{A}	23.9 ± 0.4^{AB}	16.0 ± 4.7^{B}	3.0 ± 0.5^{C}	0.3 ± 0.2^{C}	1.1 ± 0.4^{C}	1.0 ± 0.2^{C}	0.7 ± 0.3^{C}	0.9 ± 0.1^{C}
IRBB204	16.8 ± 4.4^{B}	20.3 ± 3.5^{AB}	23.0 ± 3.8^{A}	2.8 ± 0.3^{C}	1.0 ± 0.3^{C}	1.0 ± 0.6^{C}	0.9 ± 0.5^{C}	1.1 ± 0.4^{C}	0.9 ± 0.5^{C}
IRBB210	23.5 ± 4.5^{A}	26.4 ± 2.6^{A}	26.7 ± 3.7^{A}	3.5 ± 1.1^{B}	1.9 ± 0.9^{B}	0.9 ± 0.4^{B}	0.5 ± 0.1^{B}	0.8 ± 0.2^{B}	1.2 ± 0.8^{B}
IRBB211	19.7 ± 2.9^{B}	25.0 ± 2.2^{A}	23.3 ± 4.0^{A}	4.3 ± 0.9^{C}	1.3 ± 0.6^{C}	2.0 ± 0.3^{C}	2.2 ± 0.3^{C}	1.4 ± 0.3^{C}	2.1 ± 0.3^{C}
IR24	17.4 ± 5.6^{A}	15.5 ± 4.8^{A}	20.0 ± 3.5^{A}	1.6 ± 0.95^{B}	0.3 ± 0.1^{B}	0.4 ± 0.2^{B}	0.6 ± 0.7^{B}	0.4 ± 0.3^{B}	0.6 ± 0.6^{B}
IR26	15.2 ± 1.6^{A}	14.6 ± 4.3^{A}	16.5 ± 3.8^{A}	2.3 ± 0.8^{B}	0.8 ± 0.5^{B}	0.9 ± 0.4^{B}	0.7 ± 0.5^{B}	1.1 ± 0.4^{B}	1.18 ± 0.5^{B}
78-1-5	0.3 ± 0.2^{A}	0.3 ± 0.2^{A}	0.7 ± 0.3^{A}	0.5 ± 0.2^{A}	0.5 ± 0.4^{A}	0.5 ± 0.4^{A}	0.4 ± 0.4^{A}	0.3 ± 0.2^{A}	0.4 ± 0.2^{A}
BJ1	1.7 ± 0.7^{A}	2.5 ± 0.9^{A}	1.1 ± 0.5^{ABC}	0.7 ± 0.2^{BC}	1.0 ± 0.5^{BC}	0.5 ± 0.3^{BC}	0.8 ± 0.4^{BC}	0.5 ± 0.4^{C}	0.6 ± 0.4^{BC}
Asominori	21.5 ± 6.8^{A}	19.7 ± 6.7^{A}	17.3 ± 6.1^{A}	2.8 ± 1.1^{B}	2.8 ± 0.9^{B}	3.0 ± 1.0^{B}	2.1 ± 0.9^{B}	2.7 ± 1.1^{B}	2.3 ± 0.9^{B}
DV85	1.0 ± 0.7^{A}	0.9 ± 0.7^{A}	0.2 ± 0.2^{B}	0.2 ± 0.2^{B}	0.2 ± 0.2^{AB}	0.4 ± 0.3^{AB}	0.2 ± 0.1^{B}	0.6 ± 0.6^{AB}	0.2 ± 0.2^{B}
Cas209	24.7 ± 6.0^{A}	24.5 ± 2.6^{A}	24.2 ± 4.7^{A}	2.0 ± 0.8^{B}	0.8 ± 0.5^{B}	3.2 ± 0.9^{B}	0.4 ± 0.4^{B}	1.1 ± 0.9^{B}	1.0 ± 0.7^{B}
JG30	19.0 ± 3.9^{A}	23.9 ± 6.2^{A}	10.5 ± 5.2^{B}	3.8 ± 2.5^{C}	1.7 ± 0.4^{C}	1.2 ± 0.7^{C}	2.0 ± 1.3^{C}	2.2 ± 0.8^{C}	2.9 ± 1.2^{C}
CBB23	0.4 ± 0.2^{A}	0.5 ± 0.2^{A}	0.4 ± 0.1^{A}	0.3 ± 0.3^{A}	0.4 ± 0.2^{A}	0.4 ± 0.1^{A}	0.3 ± 0.1^{A}	0.5 ± 0.2^{A}	0.3 ± 0.1^{A}
DZ192	19.1 ± 1.6^{A}	19.4 ± 2.0^{A}	21.0 ± 1.0^{A}	3.2 ± 0.8^{B}	2.4 ± 0.3^{B}	4.1 ± 1.4^{B}	3.8 ± 1.2^{B}	4.4 ± 0.9^{B}	3.8 ± 1.4^{B}
Kogyoku	16.6 ± 2.2^{A}	14.7 ± 2.7^{A}	2.7 ± 0.4^{B}	$3.3.\pm1.5^{B}$	3.8 ± 0.6^{B}	1.5 ± 0.2^{B}	1.0 ± 0.2^{B}	1.3 ± 0.3^{B}	2.0 ± 0.5^{B}
X42	17.7 ± 0.6^{A}	17.2 ± 1.8^{A}	18.2 ± 0.5^{A}	5.3 ± 0.2^{B}	2.3 ± 1.3^{C}	1.8 ± 0.3^{C}	1.5 ± 0.4^{C}	1.4 ± 0.4^{C}	2.3 ± 0.2^{C}
Nipponbare	10.6 ± 1.8^{A}	10.8 ± 1.2^{A}	10.1 ± 2.1^{A}	3.3 ± 1.19^{B}	2.9 ± 0.4^{B}	2.4 ± 0.1^{B}	2.1 ± 0.2^{B}	2.3 ± 0.3^{B}	2.2 ± 0.1^{B}
Minghui63	17.8 ± 2.0^{A}	11.3 ± 2.4^{A}	15.8 ± 2.1^{A}	2.7 ± 0.3^{B}	2.3 ± 0.4^{B}	0.8 ± 0.1^{B}	0.6 ± 0.2^{B}	0.6 ± 0.2^{B}	1.7 ± 0.2^{B}
WaseAikoku3	13.1 ± 1.0^{A}	13.9 ± 0.9^{A}	13.4 ± 1.4^{A}	1.5 ± 0.4^{B}	1.4 ± 0.3^{B}	1.4 ± 0.3^{B}	1.3 ± 0.1^{B}	1.3 ± 0.1^{B}	1.5 ± 0.3^{B}

Note: Different upper letters in each row indicate significant difference in lesion lengths. Shaded is the rice variety that showed resistance to PXO99^{A} mutant (PB) with the deletion of the TALE gene cluster 3.

Supplementary Table 2. *Xa1*-mediated resistance spectrum to *X. o.* pv. *oryzae* field isolates

Country of origin	Strain	Disease reactions [a]	iTALE type A [b]	iTALE type B [b]
The Philippines	PXO61	S	+	+
	PXO71	S	+	+
	PXO79	S	+	+
	PXO86	S	+	+
	PXO99[A]	S	+	+
	PXO112	S	-	+
	PXO125	S	+	+
Republic of Korea	KXO85	R	-	-
	JW89011	R	-	+
	K202	S	+	+
Japan	T7174	R	-	+
	H75373	S	+	+
Thailand	Xoo2	S	+	-
India	A3842	S	+	+
	A3857	S	+	+
	PbXo7	S	+	-
Indonesia	IXO56	S	+	+
Nepal	NXO 260	S	+	+
Colombia	CIAT1185	S	+	+
China	ZHE 173	S	+	-
	C1	S	-	+
	C3	S	+	+
	C4	S	+	+
	C5	S	+	+
	C6	S	-	+
	C7	S	+	+
	GD1358	S	+	+
	HB17	S	+	+
	HB21	S	-	+
	HLJ72	S	-	+
	JS49-6	R	-	-
	LN57	S	-	+
	NX42	S	+	+
Australia	Aust-2013	R	-	+
	Aust-R3	R	-	+
Cameroon	AXO1947	R	-	-

[a] Disease reaction is characterized as "S" for susceptibility to bacterial infection when lesion lengths > 5 cm and resistance as "R" when lesion lengths <5 cm in *Xa1* transgenic Kitaake 12 days after inoculation.

[b] "+" and "-" denotes the presence and absence of PCR product with type-specific primers for the two types (A and B) of iTALE genes on genomic DNA from individual strains.

Supplementary Table 3. Bacterial strains used in this study

Strains or Plasmids	Relevant characteristics	Reference/ Source
**Strains**		
Xanthomonas oryzae* pv. *oryzae		
PXO99^A	Philippine race 6	(4)
PA	Tal7 and Tal8 cluster knock-out mutant of PXO99^A	This study
PB	Tal3 cluster knock-out mutant of PA	This study
PC	Tal2 cluster knock-out mutant of PB	This study
PD	Tal9 cluster knock-out mutant of PC	This study
PE	Tal5 cluster knock-out mutant of PD	This study
PF	Tal1 cluster knock-out mutant of PE	This study
PG	Tal6 cluster knock-out mutant of PF	This study
PH	Tal4 cluster knock-out mutant of PG	This study
ΔTal3	Tal3 cluster knock-out mutant of PXO99^A	This study
Xanthomonas oryzae* pv. *oryzicola		
RS105	Chinese strain	(5)
ΔTal5e	Tal5e knock-out mutant of RS105	This study
**Plasmids**		
pHZWpthXo1	pHM1 expressing *pthXo1* under lacZ promoter in pZW	(6)
pHZWtal3aC	pHM1 expressing 6126bp ClaI fragment isolated from PXO99^A containing *tal3a* in pBluescript KS(-)	This study
pHZWtal3bC	pHM1 expressing 4547bp ClaI fragment isolated from PXO99^A containing *tal3b* in pBluescript KS(-)	This study
pHZWtal3aF	pHM1 expressing *tal3a* under *lacZ* promoter with FLAG in pZW	This study
pHZWtal3bF	pHM1 expressing *tal3a* under *lacZ* promoter with FLAG tag in pZW	This study
pHZWΔ1-15	tal3a repeat-deletion derivative in pHZW	This study
pHZWΔ1-10	tal3a repeat-deletion derivative in pHZW	This study
pHZWΔ1-2	tal3a repeat-deletion derivative in pHZW	This study
pHZWtal3aFL	Full-length *tal3a* in pHZW	This study
pHZWtal3aM	tal3a with its NLS mutated in pHZW	This study
pHZWtal3aSV	tal3a with its NLS mutated and additional SV40 NLS	This study
pHZWtal3bFL	Full-length *tal3b* in pHZW	This study
pHZWtal3bM	tal3b with its NLS mutated in pHZW	This study
pHZWtal3bSV	tal3b with its NLS mutated and additional SV40 NLS	This study
pHMZWpthXo1M	PthXo1 with NLS mutated	This study
pHMZWpthXo1SV	PthXo1 with NLS mutated and additional SV40 NLS	This study
pHMZWavrXa7	AvrXa7 from pZWavrXa7 in pHM1	(1)
pHMZWavrXa7M	AvrXa7 with NLS mutated	(1)
pHMZWavrXa7SV	AvrXa7 with NLS mutated and additional SV40 NLS	(1)
pHZWXo1N-Tal3aRC	*tal3a* variant containing PthXo1 N-terminus in pHZW	This study

pHZWTal3aNR-Xo1C	*tal3a* variant containing PthXo1 C-terminus in pHZW	This study
pHZWtal4	pHM1 expressing *tal4* from PXO99^{A} in pZW	This study
pHZWtal9d	pHM1 expressing *tal9d* from PXO99^{A} in pZW	This study
pHZWtal3_PXO86	pHM1 expressing *tal3* from PXO86 in pZW	This study
pHZWtal6_PXO86	pHM1 expressing *tal6* from PXO86 in pZW	This study
pHZWtal5e_RS105	pHM1 expressing *tal5e* from RS105 in pZW	This study
pHM1tal12_BXOR1	pHM1 expressing *tal12* from BXOR1	This study
pHM1tal11h_BXOR1	pHM1 expressing *tal11h* from BXOR1	This study

Supplementary Table 4. Primers and sequence information.

Primer name	Primer Sequence (5' to 3')
Tal1F1	ATATATCTAGACGGCAGTGATGGCGAACGGTT
Tal1R1	ATATAGAGCTCTCGTTGGCCAGGCGCAGCTCG
Tal1F2	ATATAGAGCTCGCCGGCGACATCGCCCACCGC
Tal1R2	TATATGTCGACAAAGGTCCGTGCGGCATCTGG
Tal2F1	TATATCCCGGGATGCTGGCGGCCAGTA
Tal2R1	TATATAAGCTTCATGCATTCGCCGATT
Tal2F2	TATATAAGCTTCACTGCCTCCACTGCG
Tal2R2	TATATGTCGACCCACATCTGCGGCGCA
Tal3F1	ATACCCGGGCATGGCGGAATCCGGTGCG
Tal3R1	AATACTAGTAATCTTGAGAAGTTGGCCTG
Tal3F2	ATAACTAGTTCGCATGATTGATGGAGCTA
Tal3R2	TTAGCATGCTCGTACGCATGAAGGCTGGA
Tal4F1	ATATACCCGGGATGCATTTTTTGGCGAAGGGCACT
Tal4R1	ATATATCTAGAACATCCGCTGGTTGCTGCGGGCCA
Tal4F2	ATATATCTAGAGTGGACCTGCTCAAGCGAATGATG
Tal4R2	TATATGTCGACTTCTGGCGCAACTTCGGCCAGGCA
Tal5F1	ATATACCCGGGAGCAATGGCCGCATGAGCCAGG
Tal5R1	TATATGCATGCGCCGCCGCAAGCGCCGTCGGCG
Tal5F2	TGCGCCATGCATGCACTGCCTCCACTGCGGTCA
Tal5R2	TATATGTCGACCACAATCAATGGCCTGCTGGGC
Tal6F1	ATATACCCGGGATGGCAATGAGATATGGTTGAACC
Tal6R1	TATATGGATCCTCACCGCTGAAAGTGCGTGCTAAT
Tal6F2	ATATAGGATCCGATCCTGGTACGCCCATCGCTGCC

Tal6R2	TATATGTCGACCGCAGCAAGCAGCGCTTGTGGACC
Tal7/8F1	GGACCCGGGGTAGGGACCACAGACCGCTAG
Tal7/8R1	CCAAAGCTTACTGTCGAACGCACCTTCGGT
Tal7/8F2	TGGAAGCTTGACCTTGATGCGCCTAGCC
Tal7/8R2	TCCTCTAGACTGAGGCAATAGCTCCATC
Tal9F1	ATATACCCGGGATGCTCAAGAACGATCGCCTGCTG
Tal9R1	TATATGCATGCACCCGAATCCTGGGTGACACGGGC
Tal9F2	ATATAGCATGCATTTTTCACCACTTCTGAGAAGCG
Tal9R2	TATATGTCGACCCTTGCCGAGAGTTCAAGACCTGG
Tal3aHFF	CGTTGGCCGCGTTGACCAACGACCACCTCGT
Tal3aHFR	TATAAGCTTCACTTATCGTCATCGTCCTTGTAATCGGACCGTTTACGTCTGCTTG
Tal3bHFF	CGTTGGCCGCGTTGACCAACGACCAACTCGT
Tal3bHFR	TATAAGCTTCACTTATCGTCATCGTCCTTGTAATCATCATGCGATTTCCTCTTCCTTGAAT
TAL3aMF1	CAGGCATCAGGGACGGCAGCTGCCGCGGCATCCCCTACTTCAGCTCAGAC
TAL3aMF2	CACCGAATTCGAAGCCCGCTGCGGAACTATCCCCCCAGCCTCGCAGCGTTGGGACCGTA TCCTCCAGGCATCAGGGACG
TAL3aMR	ATAAGCTTCACTTATCGTCATCGTCCTTGTAATCTGCCGCGGCAGCTGCGCTTGCCCGCG TCTGATCTC
Tal3aSVR	TATAAGCTTCTAGATCAGACCTTGCGCTTCTTCTTTGGTCGCTTATCGTCATCGTCCTT
Tal3HincIIF	GTTGGCCGCGTTGACCAACGACCA
Tal3bMR	ATAAGCTTCACTTATCGTCATCGTCCTTGTAATCATCATGCGACGCGGCAGCTGCTGAATG CGGG
Tal3bSVR	AAGCTTCAGACCTTGCGCTTCTTCTTTGGTCGCGATGAGGATCCCTTATCGTCATCGTC
GFPKpF	CACCGGTACCGTCGCCACCATGGTGAGCAAGGG
GFPBamR	CCGGATCCTCCGGACTTGTACAGCTCGTC
Tal5RSF1	CGACCCGGGGCACCCGTGTCACG
Tal5RSR1	ATGGATCCTGGCGCATCGCCATCGCCGCTATGG
Tal5RSF2	TGGGATCCATCAGGCATACCTCTTTGGAGAA
Tal5RSR2	ATGTCGACTCATGCTGCACACCAAGCCGTGG
BXOR1F	CGGAGGGGTTGGATCCTACGACACGCATCGGTAGATCTG
BXOR1R	CGAGGGCCCGGGATCCGTCGCTCAGATAGTCCCCCGA
Tal3aF1	CAGACGTAAACGGTCCT
Tal3aR1	ACGCTGCCAGGTCGGCAACC
Tal3aF2	ATCAGGCGTCTTTGCATGC
Tal3aR2	TGACAGCACGATCCGATCA
Tal3bF1	GACGTCCTGCCCCGCATT
Tal3bR1	GGACGTCGCTCAGATAGTC
16SrRNA-F	TGGTAGTCCACGCCCTAAACG
16SrRNA-R	CTGGAAAGTTCCGTGGATGTC
Xa1F1	TGATTACGAATTCGAGCTAACAACTTTTCTTTTTCTGAATC
Xa1R1	TCATTACCAAAAGCATGCACTTTAAATAGTGA
Xa1F2	TGGTCACTATTTAAAGTGCATGCTTTTGGTAA

Xa1R2	TAGAGGATCCCCGGGTACCGTGACAATGCATTGGAGCGGATT
Xa1F3	AACTGATTACTCGGTGGCTTG
U	TGTAAAACGACGGCCAGT
PR1F	CGTCTTCATCACCTGCAA
PR1R	TCAGCGTACGATAGTAGTA
PBZ-F	CTCAAGATGATCGAGGAC
PBZ-R	CGTCTTCATCACCTGCAA
POX-F	ACGACATAAACGGGCCAC
POX-R	AGGTGCTAATGCCATGGCT
Actin-F	CTCAGCACATTCCAGCAGAT
Actin-R	ACAGATAGGCCGGTTGAAAA

References cited

1. B. Yang, W. Zhou, L. B. Johnson & F. F. White, The virulence factor AvrXa7 of *Xanthomonas oryzae* pv. *oryzae* is a type III secretion pathway-dependent nuclear-localized double-stranded DNA-binding protein. *Proc. Natl. Acad. Sci. U. S. A.* **97**, 9807-9812 (2000).

2. Livak, K. J. & Schmittgen, T. D. Analysis of relative gene expression data using real-time quantitative PCR and the 2(-Delta Delta C(T)) Method. *Methods* **25**, 402-408 (2001).

3. Wang, X. *et al*. Histone lysine methyltransferase SDG8 is involved in brassinosteroid-regulated gene expression in Arabidopsis thaliana. *Mol. Plant.* **7**, 1303-1315 (2014).

4. C. M. Hopkins, F. F. White, S. H. Choi, A. Guo & J. E. Leach, Identification of a family of avirulence genes from *Xanthomonas oryzae* pv. *oryzae*. *Mol. Plant Microbe Interact.* **5**, 451-459 (1992).

5. Z. Song *et al.*, Identification and Characterization of Two Novel DSF-Controlled Virulence-Associated Genes Within the nodB-rhgB Locus of *Xanthomonas oryzae* pv. *oryzicola* Rs105. *Phytopathology*. **105**, 588-596 (2015).

6. B. Yang & F. F. White, Diverse members of the AvrBs3/PthA family of type III effectors are major virulence determinants in bacterial blight disease of rice. *Mol. Plant Microbe Interact.* **17**, 1192-1200 (2004).
